# What Should We Do About Habitual Caffeine Use in Athletes?

**DOI:** 10.1007/s40279-018-0980-7

**Published:** 2018-09-01

**Authors:** Craig Pickering, John Kiely

**Affiliations:** 1 Institute of Coaching and Performance, School of Sport and Wellbeing, University of Central Lancashire, Preston, PR1 2HE UK; 2 Exercise and Nutritional Genomics Research Centre, DNAFit Ltd, London, UK

## Abstract

Caffeine is a well-established ergogenic aid, demonstrated to enhance performance across a wide range of capacities through a variety of mechanisms. As such, it is frequently used by both athletes and non-athletes alike. As a result, caffeine ingestion is ubiquitous in modern society, with athletes typically being exposed to regular non-supplemental caffeine through a variety of sources. Previously, it has been suggested that regular caffeine use may lead to habituation and subsequently a reduction in the expected ergogenic effects, thereby blunting caffeine’s performance-enhancing impact during critical training and performance events. In order to mitigate this expected performance loss, some practitioners recommended a pre-competition withdrawal period to restore the optimal performance benefits of caffeine supplementation. However, at present the evidence base exploring both caffeine habituation and withdrawal strategies in athletes is surprisingly small. Accordingly, despite the prevalence of caffeine use within athletic populations, formulating evidence-led guidelines is difficult. Here, we review the available research regarding habitual caffeine use in athletes and seek to derive rational interpretations of what is currently known—and what else we need to know—regarding habitual caffeine use in athletes, and how athletes and performance staff may pragmatically approach these important, complex, and yet under-explored phenomena.

## Key Points:

**Table:** 

Regular caffeine use appears to reduce caffeine’s ergogenic effects.
However, this reduction can be offset by an increased caffeine dose.
There appears to be no advantage, and indeed some potential disadvantages, associated with short-term pre-competition caffeine withdrawal.

## Introduction

Caffeine (1,3,7-trimethylxanthine) is a widely used drug, consumed daily by 80% of the world’s population [1]. This large-scale consumption is driven by cultural and societal trends, along with the impact of both caffeine and coffee on short-term function and long-term health. As an illustration, a number of meta-analyses have indicated the beneficial effects of caffeine on a range of chronic conditions, including cardiovascular and metabolic disorders, cancers, neurological conditions, and overall mortality [2]. Caffeine also has an acute positive effect on alertness [3], fatigue reduction [4], concentration [5], and pain perception [6]. Consequently, caffeine is widely and frequently consumed, through coffee and other caffeinated mediums, for a variety of reasons, with an estimated UK adult mean daily caffeine intake of ~ 130 mg [7].

Additionally, caffeine enhances exercise performance [8], showing ergogenic effects on aerobic endurance [9], high-intensity efforts [10], muscular endurance [11, 12], sprint performance [13], and maximum strength [14]. As a result, it is frequently used by sportspeople, with research suggesting 75–90% of athletes consume caffeine before or during competitive events [15–17]. Although average figures are not available, athletes may consume greater quantities of caffeine than their non-athletic peers due to the use of caffeinated sports supplements [18, 19]. Additionally, within caffeine experiments, trained subjects typically self-report habitual daily consumptions greater than 300 mg [20–23], demonstrating increased caffeine intakes in these populations.

Caffeine intake has a long history of use as a performance-enhancement strategy for competition, but more recently has attracted attention as a method to support training goals [24]. For example, athletes commonly use caffeine to offset fatigue associated with regular training [25, 26] and to mitigate sleep disruption caused by early morning training sessions [27] and jet lag [28]. Additionally, caffeine’s analgesic qualities may mask soreness following high training or competition loads [29–31], thereby providing an additional avenue for performance enhancement. As such, whilst caffeine use in athletes and non-athletes is conventionally considered separately, evidently there is considerable overlap between the two. This is illustrated by research investigating the optimal caffeine dose to enhance performance; historically, this relied on doses between 6 and 9 mg/kg, and sometimes as high as 13 mg/kg [32]. Such doses are difficult to attain without targeted supplementation. However, more recent research has shown caffeine can exert ergogenic effects at lower doses, including < 3 mg/kg [33]; such a dose is readily achieved through caffeine ingestion behaviors commonly seen in daily life.

Various sources contribute to the total caffeine load consumed by athletes. The most popular is coffee, with 2.25 billion cups consumed daily world-wide [34], along with other caffeinated beverages. Caffeine intake may also occur through the consumption of caffeine-containing foods, such as dark chocolate, and medications. Recently, the availability of caffeine-containing supplements, including tablets and energy drinks, has likely contributed to increased caffeine intakes in non-athletes and athletes [35]. The majority of these caffeine sources also include other compounds with potential positive effects, such as taurine in energy drinks (performance enhancement [36]), and both chlorogenic acid (health benefits [37]) and caffeic acid (promotion of post-exercise glycogen synthesis [38]) in coffee.

Caffeine’s ubiquity raises the possibility that regular, repeated exposure may blunt the subsequent physiological effects of supplementation. In animal studies, chronic caffeine intake increases adenosine receptor concentration [39, 40], attenuating caffeine’s stimulatory effects [41]. Chronic caffeine use also blunts some of the physiological responses exhibited by caffeine-naïve individuals following acute caffeine ingestion, such as increased adrenaline secretion [42]. However, caffeine habituation appears somewhat reversible following short-term cessation of caffeine consumption [43]. As such, if habitual caffeine use does blunt caffeine ergogenesis, this suggests short-term caffeine withdrawal may be advantageous to habitual caffeine users. At present, advice given to athletes regarding caffeine habituation and withdrawal is often vague and conflicting. The International Society of Sports Nutrition’s position stand summarizes research pertaining to caffeine habituation [44], but stops short of making recommendations. Contemporary sources further this confusion; some [45] state that habituation potentially harms performance, recommending pre-competition withdrawal. Others suggest habituation does not reduce caffeine’s ergogenic benefits, rendering pre-competition withdrawal unnecessary [46]. Consequently, athletes receive confusingly mixed messages, and best practice remains unclear.

The aim of this paper, therefore, is to reflect on two unresolved questions, both directly relevant to athletes seeking to optimize pre-competition caffeine use. First, we consider whether regular caffeine intake alters its ergogenic effects. Second, we explore whether, in habitual users, short-term pre-competition caffeine withdrawal restores caffeine’s ergogenic benefits. Both are critical considerations for athletes seeking to develop caffeine supplementation strategies to augment performance. Does regular caffeine intake alter caffeine’s ergogenic properties at the time when an athlete may need them most—during competition—or does caffeine habituation not impact performance?

## Does Chronic Caffeine Use Alter Caffeine’s Acute Ergogenic Effects?

### The Problem with Assessing Habitual Caffeine Use

Whilst tempting to believe the caffeine content of various caffeine sources is stable and consistent, there is considerable variation in concentrations, both between different caffeine sources and within the same source across different time points [47, 48]. For example, the caffeine content of a single serving of espresso can vary from 25 to 214 mg across locations and by as much as 132 mg at the same location at different times [47]. Consequently, even when the same beverage is consumed on a regular basis, daily caffeine intake likely varies considerably, making accurate determination of habitual caffeine intake problematic [49]. Within studies, food/beverage frequency questionnaires are often used to quantify habitual caffeine intake. However, given the considerable variation between caffeine concentrations within the same source, these questionnaires likely do not accurately capture true habitual caffeine intake. More objective measures of ingestion, such as urinary caffeine output [50] or plasma caffeine (or metabolite) levels [51], do exist, and could be used to more accurately quantify habitual caffeine intake alongside validated caffeine intake questionnaires, although clearly such methods pose their own set of logistical challenges. Such variation in caffeine concentrations across sources and time is also problematic for athletes trying to accurately quantify desired caffeine doses.

Additionally, recent evidence suggests habitual caffeine use is influenced by individual responsiveness to its physiological actions. For example, single-nucleotide polymorphisms (SNPs) in *ADORA2A* and *CYP1A2* appear to impact habitual caffeine intake [52–54], with those possessing the more sensitive genotype consuming less caffeine. Such findings further complicate our understanding of the effects of habitual caffeine use in athletes; given the individual responsiveness to caffeine, is habitual use a function of specific genetic polymorphisms, making measurement of habitual caffeine intake a proxy for genotype? Furthermore, these SNPs may also impact caffeine’s ergogenic effects, with C allele carriers of *CYP1A2* (rs762551) tending to exhibit smaller performance benefits [55, 56], potentially demonstrating an ergolytic effect [57]. In a pilot study exploring the impact of *ADORA2A* on caffeine ergogenesis, the TT genotype was associated with greater performance enhancement following caffeine ingestion prior to a cycle ergometer time trial [58]. A potential confounder here, however, is that CT/TT genotypes also tended to habitually consume more caffeine than TT genotypes (although this was not significant), and may subsequently have become habituated. Furthermore, the small sample size (*n* = 12) also severely limits the interpretations possible. These findings further complicate the analysis of the impact of habitual caffeine use on athletes; are we measuring the impact of habitual caffeine use on performance, or are we measuring the interaction of caffeine and genotype—which is rarely determined in investigative studies—on performance?

### Habitual Caffeine Use and Athletic Performance

Habitual caffeine intake modifies physiological and cognitive responses to acute caffeine doses [41, 42, 59]. The underpinning mechanisms are varied, and remain incompletely understood. The majority of research in this field utilizes animal models. Here, the dose of caffeine used is often very large (such as 80 mg/kg) [60], and often given as a single bolus, as opposed to the lower, more regular doses typical of human ingestion patterns, making direct comparisons difficult. Nevertheless, perhaps the best supported pathway of caffeine habituation is the alteration of physiological responses caused by the up-regulation of adenosine receptors [59]. Additionally, regular exposure to substrates that induce cytochrome P450 1A2—a key part of the caffeine metabolization pathway [61, 62] —appears to increase the induction speed of that enzyme [63, 64]. As such, habitual caffeine use may alter caffeine metabolization speed. The time frame of such changes is poorly understood, although withdrawal symptoms, suggesting habituation, can occur after only 3 days of caffeine consumption [65]. For athletes, the key consideration is whether this impacts sporting performance. Van Soeren et al. [66] examined the impact of caffeine on physiological responses in habitual users and non-users in an exercise setting, focusing on alterations in plasma epinephrine, plasma and urinary caffeine concentrations, free fatty acid concentrations, and respiratory exchange ratio, as opposed to specific performance tests. Whilst there were apparent differences between the groups in some of these variables, in the absence of performance tests, it is impossible to determine whether these differences resulted in any real-world performance detriments. Similarly, other studies also used subject pools comprising habitual users and non-users, but again did not analyze differences in performance between them [67, 68].

The first study utilizing a performance test [20] recruited 17 moderately trained males, of whom eight were non-habitual (< 25 mg/day) and nine habitual caffeine users (> 300 mg/day) (Table 1). All subjects undertook time-to-exhaustion trials under three experimental conditions: placebo, 3 mg/kg caffeine, and 5 mg/kg caffeine. There were no differences between the groups regarding time to exhaustion at either dose, indicating habitual caffeine use does not blunt caffeine’s ergogenic effects. In contrast, Bell and McLellan [22] recruited 13 habitual users (≥ 300 mg/day) and eight non-users (< 50 mg/day) to undertake cycle ergometer time-to-exhaustion tests following ingestion of 5 mg/kg caffeine either 1, 3, or 6 h prior. Subsequent results suggested the magnitude and duration of ergogenic effects were greater in the non-users than the habitual users. When caffeine was consumed both 1 and 3 h pre-test, the habitual caffeine users exhibited an ergogenic effect following caffeine ingestion, but this was smaller than that exhibited by the non-users—indicating a blunting, but not loss of, caffeine ergogenicity with habitual use.

**Table 1:** Characteristics and findings of studies directly examining the effects of habitual caffeine intake on exercise performance

Study	Subjects	Habitual dose	Pre-trial dose	Performance trial	Performance outcome
Dodd et al. [20]	17 moderately trained males	< 25 mg/day for non-habitual users; > 300 mg/day for habitual users	3 and 5 mg/kg	Graded incremental cycle ergometer: increases of 30 W every 2 min until subjects could not maintain a set power output	No difference between habitual and non-habitual users in time to exhaustion
Bell and McLellan [22]	21 male and female subjects	< 50 mg/day for non-habitual users; ≥ 300 mg/day for habitual users	5 mg/kg	Time to exhaustion cycle ergometer at 80% VO_2max_	Habitual caffeine use appeared to reduce the ergogenic effects of caffeine
Beaumont et al. [69]	18 habitually low caffeine users	3 mg/kg	3 mg/kg	60-min cycle at 60% VO_2peak_, followed by maximum work completed in 30 min	Habitual caffeine use reduced the ergogenic effects of caffeine
Gonçalves et al. [23]	40 male endurance-trained cyclists	58 mg/day (low group); 143 mg/day (moderate group); 351 mg/day (high group)	6 mg/kg	Cycle ergometer time trial; set amount of work in shortest possible time	No effect of habitual caffeine intake

*VO*_*2max*_ maximal oxygen consumption, *VO*_*2peak*_ peak oxygen consumption

More recently, 18 habitually low (< 75 mg/day) caffeine users were randomly assigned to receive either daily caffeine supplementation (starting at 1.5 mg/kg and increasing to 3 mg/kg) or placebo for 28 days [69]. Following this sustained supplementation period, all subjects ingested 3 mg/kg caffeine 60 min prior to a 60-min cycle at 60% of peak oxygen consumption (*V*O_2peak_), immediately followed by a 30-min maximum cycle. When compared to their pre-intervention, caffeine-supplemented exercise trial, there was a 7.3% reduction in total work produced by habituated caffeine users, representing a *probably harmful* effect of habitual caffeine use based on magnitude-based inferences. However, the ergogenic effect was not completely diminished; compared to the pre-intervention placebo trial, caffeine supplementation still exhibited a *possibly beneficial* effect following acute caffeine ingestion. In contrast, the chronic placebo supplementation group exhibited no differences between the pre- and post-intervention caffeine trials, again suggesting that regular caffeine use reduces, but does not eliminate, caffeine’s ergogenic effects.

Finally, Gonçalves and colleagues [23] stratified 40 male endurance-trained cyclists into groups based on their habitual caffeine intake: low (mean = 58 mg/day), moderate (mean = 143 mg/day), and high (mean = 351 mg/day). These subjects underwent a simulated cycling time trial of approximately 30 min duration under three conditions: a caffeine trial with 6 mg/kg, a placebo trial, and a control trial. Habitual caffeine intake exerted no influence on exercise performance, suggesting caffeine habituation had no detrimental impact on caffeine ergogenesis.

### What to Make of These Results?

Gonçalves et al. [23] titled their paper “Dispelling the myth that habitual caffeine consumption influences the performance response to acute caffeine supplementation.” Whilst this title certainly reflects their study results, it is perhaps premature given the results of Bell and McLellan [22] and Beaumont et al. [69]. Dispelling “the myth” requires more than a single contradictory finding, especially given recent findings regarding differences in ergogenic effects in habitual and non-habitual caffeine users following low-dose caffeine-gum supplementation [70]. In evaluating this claim, it is worth inspecting the differences in study designs. The major distinction lies in the different habitual and pre-trial caffeine doses employed. Beaumont et al. [69] utilized habitual and pre-trial caffeine doses of 3 mg/kg, such that the pre-trial and habitual caffeine doses were identical. Bell and McLellan [22] utilized a pre-trial dose of 5 mg/kg. The subjects in the habitual caffeine group had habitual intakes of at least 300 mg/day, equating to approximately 4 mg/kg/day. In this case, the pre-trial caffeine dose was close to, but slightly greater than, the habitual caffeine dose. In contrast, in Gonçalves et al.’s study [23], habitual caffeine intake in the high group was 351 mg/day, equating to ~ 4.5 mg/kg/day, somewhat lower than the pre-trial dose of 6 mg/kg. Dodd et al. [20] did not provide body mass data, so we were unable to analyze differences between habitual and pre-trial caffeine dose; however, with a mean caffeine intake of 495 mg/day in the habitual group, it is unlikely that the pre-trial dose of 5 mg/kg was substantially greater than this.

The studies are also heterogeneous in other aspects. The exercise trials were a mix of time-to-exhaustion [20, 22] and time trials [23], consequently impacting the ability to directly compare trials [71]. Whilst all studies recruited males, Bell and McLellan [22] also recruited females, who may metabolize caffeine differently to males [72, 73], although it is not clear whether this impacts caffeine ergogenesis [74, 75]. Additionally, training status varied between studies, with competitive cyclists [23] and recreationally active subjects [69] recruited. Again, training status potentially alters the ergogenic effects of caffeine [76, 77]. Furthermore, differences in pre-trial fasting (e.g., overnight [69] vs 4 h [20]) may have impacted both energy status and caffeine ergogenicity. Finally, as previously discussed, habitual caffeine intake was assessed via food frequency questionnaires, which are subject to recall bias [78], further hampering thorough interpretation.

In summary, it appears that habitual caffeine use may reduce the magnitude of caffeine’s ergogenic effects; however, any reductions in caffeine ergogenicity with habitual use are potentially modified by pre-trial doses substantially greater than habitual intake. At present, these conclusions are based on little evidence, and further research is certainly required.

### What Does This Mean for Athletes?

If caffeine-habituated individuals require greater caffeine doses to maintain ergogenic effects, this is potentially problematic for athletes. Evidence suggests there are no additional ergogenic effects of caffeine above doses of 9 mg/kg [32, 79]; if athletes habitually consume moderate-to-high doses of caffeine (~ 4–5 mg/kg), there may be a point at which further caffeine ingestion does not restore caffeine’s full ergogenic potential. Whilst such habitual intakes appear extreme, the regular intake of subjects in some research studies [80] reaches this level. Additionally, higher doses of caffeine may increase the risk of reported side effects, such as tremor, insomnia, and increased heart rate [32, 79, 81].

Furthermore, as caffeine’s ergogenic effects occur through a variety of mechanisms, the time course and magnitude of habituation likely vary for each molecular pathway. For example, caffeine is primarily metabolized by cytochrome P450 enzymes [62]. Regular caffeine consumption upregulates production of this enzyme group [82], increasing metabolization speed in habitual users. Thus, variation in cytochrome P450 activity, which is partially genetically determined [61], may impact caffeine ergogenesis [55–57]. This increased caffeine metabolization rate in habitual users is likely a major reason why regular use may require larger dosages to exert an effect previously experienced at lower dosages, with greater amounts of caffeine required to maintain the concentration driving the ergogenic effects. Aside from metabolization speed, habituation may also occur through changes in adenosine receptor density [41], which again is subject to inter-individual variability mediated through genetic variation [58]. The time course of habituation is poorly understood, potentially differs between pathways, and is likely affected by factors such as habitual dose [60]. For example, in rats, complete caffeine tolerance occurs following a dose of 40 mg/kg spread over the preceding 24 h [83], whilst a lower dose (7.5 mg/kg) requires 14 days to achieve habituation [41]. Additionally, the time course for habituation within each individual pathway may also differ between individuals, adding further complexity to our attempts to understand the effects of caffeine habituation in athletes.

## Does Caffeine Withdrawal in Habitual Users Restore Optimized Ergogenic Effects?

If habitual caffeine use requires an increased pre-competition dose to restore the ergogenic effects, and this increase enlarges the risk of negative side effects, an alternative option may be for the athlete to cease caffeine use for a short period beforehand. This method has previously been proposed as a way to become re-sensitized to caffeine’s physiological effects [80], potentially mediated through changes in adenosine receptors [84] or by overcoming compensatory changes in the dopaminergic system due to chronic and consistent blockage of adenosine receptors with high caffeine use [85]. The time course of re-sensitization remains unclear, and is potentially impacted by both the duration and extent of prior caffeine exposures [65]. Similar to caffeine habituation studies, many studies exploring caffeine withdrawal are conducted in rodents, utilizing what would be, for humans, unrealistically large caffeine dosages (e.g., 194 mg/kg/day) [84]. Finally, it remains unclear whether caffeine withdrawal actually re-sensitizes subjects to the physiological effects of caffeine, or whether the reintroduction of caffeine following a period of abstinence merely reverses the many side effects associated with withdrawal [86–88]. Again, despite its relevance, only a few studies have investigated the potential association between temporary caffeine withdrawal and the restoration of caffeine’s ergogenic effects.

An early study [80] examined the influence of 4 days’ caffeine withdrawal on the ergogenic effects of 5 mg/kg of caffeine in six habitual (> 600 mg/day) caffeine users (Table 2). Whilst the withdrawal period increased the sensitivity of some individuals to caffeine in terms of physiological responses, physical performance itself was not measured. Subsequently, Van Soeren and Graham [21] put six habitual caffeine users (mean daily caffeine intake 761 mg/day) through time-to-exhaustion cycling trials with 0-, 2-, and 4-day caffeine withdrawal periods prior to a pre-trial caffeine dose of 6 mg/kg. Although time to exhaustion was longer in all caffeine trials compared to placebo, no significant differences existed between withdrawal periods.

**Table 2:** Characteristics and findings of studies directly examining the effects of caffeine withdrawal on exercise performance

Study	Subjects	Habitual dose	Washout period	Pre-trial dose	Outcome
Fisher et al. [80]	6 habitual users	> 600 mg/day	4 days	5 mg/kg	No direct performance measure
Van Soeren and Graham [21]	6 habitual users	Mean of 761 mg/day	0, 2, and 4 days	6 mg/kg	No significant differences in time-trial performance between washout periods
Irwin et al. [89]	12 well-trained male cyclists	Mean of 240 mg/day (range 18–469 mg/day)	4 days (placebo or 1.5 mg/kg)	3 mg/kg	No difference between placebo and caffeine group

More recently [89] 12 well-trained male cyclists with an average habitual caffeine intake of 240 mg/day abstained from caffeine for 4 days, during which they received daily supplementation with either placebo or 1.5 mg/kg caffeine. On the fifth day, the subjects consumed 3 mg/kg caffeine 90 min prior to a cycle time trial. Pre-trial caffeine ingestion significantly enhanced time-trial performance in both conditions (habitual placebo and caffeine), with no significant differences between the two. Consequently, the authors concluded the 4-day withdrawal period did not enhance caffeine ergogenicity. As with habituation, studies exploring caffeine withdrawal are heterogeneous, with differences in subject sex, habitual caffeine intakes, and exercise modalities. Additionally, the findings from Fisher et al. [80] and Van Soeren and Graham [21] may have been affected by very high habitual intakes, which were over four times the average population daily intake [7] and double the doses commonly seen in habitual users in caffeine studies [20]; as such, it is difficult to extrapolate these results into the context of caffeine utilization in athletes.

In summary, it appears short-term, pre-competition caffeine withdrawal in habitual users does not enhance caffeine’s ergogenic effects. Withdrawal is also associated with numerous negative outcomes, including headaches, fatigue [21], irritability, muscle pain, sleep disturbances, and nausea [85, 87]. Fortunately, such symptoms appear limited to a sub-set of individuals, are typically mild and temporary [24], and reverse with caffeine ingestion [88]. Nevertheless, such acute withdrawal symptoms, close to key competitions, may negatively affect athlete subjective confidence and well-being.

## Rational Interpretations and Practical Implications

The summarized research (Tables 1 and 2) suggests:

- Habituation, in terms of reduced ergogenic effects, may occur in those who regularly consume caffeine [69].
- Habituation can result in reduced caffeine ergogenesis, but this may be offset if the pre-competition caffeine dose is greater than the habitual dose [23].
- Short-term caffeine withdrawal prior to key events appears to offer little or no competitive benefit to habitual users [89].

With caffeine shown to exert its greatest ergogenic effects at between 3 and 9 mg/kg, there is a wide range of dosages for athletes to consume around competition. Previously, we discussed the considerable inter-individual variation underpinning both caffeine ergogenicity and the dose necessary to achieve performance benefits [90]. Such variation is a product of complex interactions between genetics, environmental exposures, and epigenetic modifications. Accordingly, any one-size-fits-all recommendations regarding caffeine use appear fundamentally flawed, and should be avoided. Some individuals may find the peak ergogenic effects of caffeine occur towards the lower end of this 3- to 9-mg/kg window; here, it may be prudent to habitually consume lower caffeine doses, as the scope for consuming the required caffeine dose to offset habituation is smaller. Conversely, individuals exhibiting peak ergogenic benefits at higher doses may tolerate greater habitual intakes, as there is an increased scope for consumption of greater pre-competition caffeine doses. A further consideration is that of the negative side effects of caffeine use, which tend to be most pronounced in non-habitual users [32, 79, 81]. In this case, if the athlete wishes to explore the potential ergogenic benefits of caffeine, then initiating habitual use may be beneficial in order to reduce the prevalence or intensity of any negative side effects.

### Inter-individual Variation in Caffeine Habituation and Performance

Considerable variation exists in caffeine responsiveness between individuals [90]. This inter-individual variation is partially genetically determined, with SNPs in genes such as *ADORA2A* and *CYP1A2* influencing both habitual caffeine use [52, 53] and caffeine ergogenicity [55–58]. As such, further insights into the genetic differences between subjects may allow for a greater individualization of pre-competition caffeine advice [90]. Adding to this complexity, there is likely inter-individual variation in the time-course, magnitude, and mechanisms of caffeine habitation. For example, caffeine’s ergogenic effects are mediated via multiple proposed pathways, such as altering fat metabolism [91, 92], or reducing perceived exercise-associated pain [93]. Genetic variations within these pathways, coupled with individual history, may modify the effectiveness of each pathway, altering the performance of individuals to differing extents. Subsequently, for example, during a marathon, athlete A may gain a greater performance benefit from caffeine’s impact on fat metabolism, whilst athlete B gains their ergogenic effect from a reduction in pain. Does habitual caffeine use alter both these phenotypes to the same extent? Does regular caffeine exposure modify fat metabolism to a greater or lesser extent than pain reduction? Can the diminished ergogenic effect be offset through other ergogenic aids, such as paracetamol in the case of pain reduction [94]? Does habituation occur sooner in one pathway than another? Do the multiple ergogenic benefits of caffeine manifest in all consumers or is every individual specifically sensitive to some while resistant to others? The answers to these questions are undoubtedly complex, and we may never know the answers—illustrating how wary we should be of one-size-fits-all advice.

### Some Tentative Recommendations

Given the current lack of adequate research and the inevitability of inter-individual variations in response to caffeine, any advice at this stage is somewhat speculative. Additionally, a wide-ranging assessment of caffeine recommendations is beyond the scope of this review; however, based on the limited research available, when considering caffeine habituation and pre-competition exercise withdrawal, it seems sensible to suggest the following:

1. As caffeine is widely consumed, with a number of social and health benefits outside of performance enhancement, a pragmatic approach is to understand that the majority of athletes will consume caffeine outside of deliberate pre-competition ingestion. Based on the evidence presented here, it appears moderate (~ 3 mg/kg) daily doses of caffeine will not be problematic for most athletes, most of the time.
2. As caffeine has a multitude of positive effects on training performance, it can—and perhaps should—be used either prior to or during training. For most sport types, if used prior to training, we would recommend consuming caffeine approximately 60 min prior to the onset of the first working set of the session. This time scale will differ between individuals [95], event types [44], and methods of caffeine ingestion, with caffeinated mouth rinses and gums often requiring far less time [96]. If used during prolonged training sessions, there is evidence that later ingestion of caffeine, and at lower doses, may be effective [97].
3. For most athletes, the total of regular caffeine intake spread across the day, including the pre- and intra-training dose, should not exceed 3 mg/kg, as this will increase the required pre-competition caffeine dose substantially. Ingesting caffeine later within a training session often requires a lower dose [97], which may further guard against habituation.
4. Given the individual response to caffeine, both in terms of habituation and ergogenesis, athletes should experiment with various doses and timing strategies when using caffeine to enhance performance [90, 95]. As a broad start point, we recommend athletes who are not caffeine naïve, but also not high habitual users, utilize approximately 3 mg/kg approximately 60 min prior to a competitive bout, and then adjust accordingly. Alternatively, athletes may wish to estimate their daily caffeine intake (whilst understanding the methodological issues in doing so), and double this dose to obtain an idea of their pre-competition dose. For athletes competing in prolonged events, caffeine may need to be consumed closer to, or indeed within, the competitive bout in order to enhance performance.
5. There appears to be no benefit from, and potentially negative consequences of, a short-term, pre-competition caffeine withdrawal period. The impact of longer (> 7-day) withdrawal periods has not, to our knowledge, been explored in the context of performance.
6. The impact of pre-competition caffeine doses on a subsequent performance bout in a short time frame (such as in heats and finals of a track event separated by ~ 90 min) is currently poorly explored [98]. Again, a pragmatic approach is perhaps required; if the athlete requires the performance benefits of caffeine to progress through the first event, then caffeine should be consumed at that time point. Athletes may then experiment with the optimal “top-up” dose to be consumed between heats and final.

Future research should seek to confirm whether habituation systematically reduces caffeine’s ergogenic effects (as per Beaumont et al. [69]), confirm whether increased caffeine dosages can offset this reduction (as per our analysis of Gonçalves et al. [23]), and elucidate the drivers of the inter-individual response to each. In addition, investigating the impact of longer term (> 7-day) caffeine withdrawal periods, to see if this enhances the ergogenic effect of a subsequent caffeine dose, seems advisable. Such an approach may prove useful to those competing infrequently, such as in a marathon, as opposed to those competing on a more regular basis, where constant exposure to caffeine would be expected. Finally, the impact of caffeine ingestion on sequential competitive bouts performed in a short time frame remains, from the athlete’s perspective, highly relevant, and yet is poorly explored [96]. Further exploration of this would likely be of great practical use to athletes.

## Conclusions

In summary, caffeine habituation may blunt the expected ergogenic effects, although this may be mitigated by increasing pre-competition caffeine dose. Furthermore, short-term pre-competition caffeine withdrawal appears to offer little benefit, and given the potential negative side-effects, such practices are not recommended. Currently, it appears that moderate (~ 3 mg/kg/day) chronic caffeine intake is not problematic for most athletes. However, there is likely to be substantial inter-individual variation [95], both in regard to optimal caffeine dose and timing and also the effects of habituation and withdrawal. As such, we should be wary of a one-size-fits-all approach. In the absence of well-replicated research in this area, a pragmatic approach is recommended for athletes. Here, the currently available research provides athletes and practitioners with an informed starting point, from which they can then experiment with different caffeine strategies—both acute and chronic—during training periods in order to enhance caffeine’s ergogenic effects within competition. Once an effective strategy is found, it can be refined and rehearsed, optimizing performance. As illustrated, the relationship between caffeine, habitual use, and performance is complex and nuanced, poorly understood, and ever-changing within the individual. Nevertheless, conventionally, we persist in recycling and utilizing generalized guidelines to inform caffeine ingestion strategies. We can, and should, improve this process.
